# Modelling of Rod-Like Fillers’ Rotation and Translation near Two Growing Cells in Conductive Polymer Composite Foam Processing

**DOI:** 10.3390/polym10030261

**Published:** 2018-03-02

**Authors:** Sai Wang, Amir Ameli, Vahid Shaayegan, Yasamin Kazemi, Yifeng Huang, Hani E. Naguib, Chul B. Park

**Affiliations:** 1 Microcellular Plastics Manufacturing Laboratory, Department of Mechanical and Industrial Engineering, University of Toronto, Toronto, ON M5S 3G8, Canada; swang@mie.utoronto.ca (S.W.); vahidsh@mie.utoronto.ca (V.S.); yasamin@mie.utoronto.ca (Y.K.); 2 Advanced Composites Laboratory, School of Mechanical and Materials Engineering, Washington State University Tri-Cities, Richland, WA 99354, USA; a.ameli@wsu.edu; 3 Drinking Water Research Group, Department of Civil Engineering, University of Toronto, Toronto, ON M5B 1A4, Canada; yf.huang@utoronto.ca; 4 Smart and Adaptive Polymer Laboratory, Department of Mechanical and Industrial Engineering, University of Toronto, Toronto, ON M5S 3G8, Canada; naguib@mie.utoronto.ca

**Keywords:** conductive filler, orientation, conductive polymer composites, foam, model

## Abstract

We developed a simple analytical model to describe the instantaneous location and angle of rod-like conductive fillers as a function of cell growth during the foaming of conductive polymer composites (CPCs). First, we modelled the motion of the fillers that resulted from the growth of one cell. Then, by taking into account the fillers located at the line that connected the centres of the two growing cells, we found the final filler’s angle and location. We identified this as a function of the corresponding cell size, filler size, and the filler’s initial angle and location. We based the model’s development on the assumption that a polymer melt is incompressible during cell growth. The two-cell growth model is better than the one-cell growth model because it describes the filler’s movement in the cell wall between the two growing cells. The results revealed that the fillers near the cell were the ones most affected by the cell growth, while those at the midpoint between the two cells were the least affected. As a cell grows, its affected polymer area also increases. A dimensionless factor η was introduced to demonstrate the effects of the cell size and the filler length on the filler’s interconnectivity in the CPC foams. It is vital to keep the filler length comparable to the cell size when preparing CPC foams with the desired electrical conductivity. Our research provides a deeper understanding of the mechanism through which foaming influences the filler connections in CPC foams.

## 1. Introduction

In past decades, conductive polymer composites (CPCs) have been of great interest to those developing new generations of materials with unique properties and functions [1,2,3,4] and showed great potential for use in various applications such as high-dielectric materials for charge storage [5,6,7,8], electromagnetic interference (EMI) shielding [9,10,11,12], and bipolar plates of fuel cells [13,14,15]. The electrical conductivity can be easily established by adding micro- or nano-size conductive additives (that is, stainless-steel fiber [16], graphene [17,18,19], carbon fiber [20], carbon nanofiber [21,22,23], carbon black [4,24], and carbon nanotubes [25,26,27]) to the polymer matrix.

Efforts have been made to obtain a high electrical conductivity at low filler loadings [28,29,30,31]. Recent foaming technology, such as foam injection molding [32] and batch-type foaming [33], has shown promise in decreasing the electrical percolation threshold and increasing the CPC’s conductivity. Foam injection molding can enhance the electrical conductivity in the through-plane direction because the preferentially aligned fillers, which have a machine-direction and/or in-plane orientations due to the shear force during the injection process, can be rotated toward the through-plane direction by the biaxial stretching in the polymer matrix caused by cell growth [34,35,36].

Unlike the foam injection molding, the initial filler distribution of the solid precursors in batch foaming can be controlled so as to be random. Previous research shows that cell growth may then slightly align the fillers, and thus may increase the possibility of filler connections, which ultimately will help to establish conductive networks [37,38]. However, Rizvi et al. [39] and Sun et al. [40] both found that the electrical conductivity of CPC foams was lower than their solid precursors. It is unknown whether these contradictory experimental results occurred because the optimal foaming and material conditions were only applied in some cases or because other factors, such as the polymer matrix’s crystallinity, also influenced the conductivity [41]. Nevertheless, it remains clear that cellular growth, either in injection molding or in batch foaming, significantly affects the filler alignment in CPC foams. It is thus important to comprehend the correlations between the cellular growth and the filler movement. This will eventually make it possible to estimate the filler orientation’s final state based on its initial orientation status and on the foaming process. This will result not only in a fundamental understanding of the foaming action effect on the electrical conductivity, but it will also help with the systematic optimization of the foaming processes, which will yield products with enhanced electrical properties. 

Shaayegan et al. [20] developed a visualization system to observe the instantaneous fiber movement (i.e., rotation and translation) as a function of the corresponding cell size and of the fiber’s initial orientation and location. However, their work was limited to the growth of one cell. The effect of multi-cell growth on the filler’s motion has more importance in terms of practical applications.

Therefore, in our study, we introduced an analytical model to describe the filler’s rotation and translation during cellular growth in polymer composite foams. Using this model, we have attempted to elucidate the effects of multiple factors, including the cell size, the filler length, and the filler’s relative position with respect to the cell nucleus, on the displacement and re-orientation of conductive fillers. By analyzing the rotation and translation of the rod-like fillers in the cell walls during cell growth, we were able to better understand how cellular growth affects the filler’s angle and location in real CPC foam. This would enable us to demonstrate the feasibility of foaming as a potential strategy to decrease the percolation threshold in CPCs.

## 2. Theoretical Estimation

This paper is about cell growth and how its deterministic modelling affects the movements of various rod-like fillers. First, we focused on how any filler can be moved by any one cell located close to it. We described the filler’s movements as a combination of (i) the filler’s rotation and (ii) the translation of the filler’s centre point by the growing cell. Then, we extended this singular filler/cell case to a slightly more complicated case of one filler and two cells. We asked how one filler could be moved by two growing cells located close to it. This example showed us how the filler would move if there were multiple cells present. This approach provided us a clear insight into the filler’s movement in relation to the cells growing near it. 

Then, we extended the example of a single filler’s movement to the movement of multiple fillers. Since the filler movement is independent, the one-filler’s movement can be extended to the multiple-fillers’ movement independently with respect to each other. Using this approach, we could accurately trace how all of the fillers were moving in the polymer matrix while the cells were growing.

### 2.1. One-Cell Model Approach

To model the movement of rod-like fillers during foaming, we first considered a simple case of one-cell growth. We made several assumptions prior to developing the model. In the case of one-cell growth, the following conditions apply: (1) The radial growth of the cell is symmetric; (2) The fillers are rigid and do not slip over the adjacent polymer matrix; (3) Upon cell growth, the filler only changes with respect to the line that passes through the cell centre and the filler midpoint; (4) The growth of a cell forces the filler to translate in the radial direction of the cell centre to the filler midpoint, and the filler rotates around its midpoint; (5) The filler does not interfere with the adjacent filler; (6) The fillers cannot penetrate into the cells and are always located in the polymer matrix; (7) The cell structure is a closed cell and no open cell is considered; (8) The polymer matrix is incompressible.

**Figure 1:**
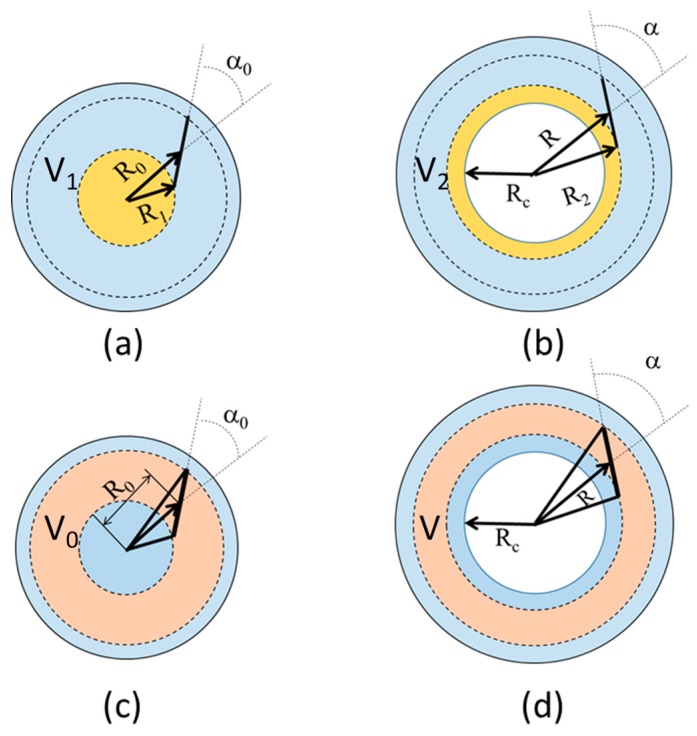
Two-dimensional (2-D) schematic illustration of filler alignment: (**a**,**c**) the initial state with an initial filler angle of α_0_ and an initial filler location of *R*_0_; (**b**,**d**) an instantaneous state with a filler angle α and a filler location *R* after expansion with a cell radius *R*_c_. White area in the sphere represents the cell. Initial and instantaneous volume of incompressible polymer sphere is used to calculate the changes in “filler angle” and “filler location”.

As Figure 1 shows, a set of two parameters was used to describe the fillers’ locations and orientations as well as their changes during cellular growth. One parameter was the “filler location”, which was defined as the radial distance of the filler midpoint, with respect to the corresponding cell centre (*R*_0_ for the initial location in Figure 1a,c and *R* for instantaneous locations in Figure 1b,d). The second parameter was the “filler angle”, which was defined as the angle of the rod-like filler axis with respect to the line passing through its midpoint and through the cell centre (α_0_ for initial angle in Figure 1a,c and α for the instantaneous angle in Figure 1b,d).

Due to the polymer’s incompressibility, the initial volume, *V*_1_, of a sphere of a radius *R*_1_ (Figure 1a) remains unchanged during cellular growth. As shown in Figure 1a, the *R*_1_ can be determined as follows:

(1) $$V_{1}=\frac{4}{3}\pi R_{1}{}^{3}$$

and from trigonometry,

(2) $$R_{1}^{2}=R_{0}^{2}+{\left(\frac{l}{2}\right)}^{2}-2\cdot R_{0}\cdot \frac{l}{2}\cos\alpha_{0}$$

where *l* is the length of the filler. The *V*_1_ can then be written as follows:

(3) $$V_{1}=\frac{4}{3}\pi {\left(R_{0}{}^{2}+\frac{l^{2}}{4}-R_{0}\cdot l\cdot \cos\alpha_{0}\right)}^{\frac{3}{2}}$$

After expansion, the polymer volume of the $V_{1}$ is transferred to a spherical ring with inner and outer radii of $R_{c}$ and $R_{2}$, having a volume of *V*_2_, as shown in Figure 1b, where *R*_c_ is the cell radius at any time. The *V*_2_ can then be written as follows:

(4) $$V_{2}=\frac{4}{3}\pi R_{2}{}^{3}-\frac{4}{3}\pi R_{c}{}^{3}$$

and from trigonometry,

(5) $$R_{2}^{2}=R^{2}+{\left(\frac{l}{2}\right)}^{2}-2\cdot R\cdot \frac{l}{2}\cos\alpha$$

Similar to the *V*_1_, the *V*_2_ can be written as follows:

(6) $$V_{2}=\frac{4}{3}\pi \left[{\left(R^{2}+\frac{l^{2}}{4}-R\cdot l\cdot \cos\alpha \right)}^{\frac{3}{2}}-R_{c}{}^{3}\right]$$

Furthermore, the volume of any spherical envelope surrounding the filler is invariable during the expansion phase because the expansion is symmetrical. This is schematically shown in Figure 1c,d, the volume of the initial and instantaneous spherical rings (envelopes) were *V*_0_ and *V*, respectively. Therefore, we can write *V*_0_ = *V*. Using trigonometric relations, they can be calculated as a function of the radii *R*_0_ and *R*, respectively, as follows:

(7) $$V_{0}=\frac{4}{3}\pi \left\{{\left[R_{0}{}^{2}+{\left(\frac{l}{2}\right)}^{2}-2R_{0}\cdot \frac{l}{2}\cos\left(\pi -\alpha_{0}\right)\right]}^{\frac{3}{2}}-{\left[R_{0}{}^{2}+{\left(\frac{l}{2}\right)}^{2}-2R_{0}\cdot \frac{l}{2}\cos\alpha_{0}\right]}^{\frac{3}{2}}\right\}$$

(8) $$V=\frac{4}{3}\pi \left\{{\left[R^{2}+{\left(\frac{l}{2}\right)}^{2}-2R\cdot \frac{l}{2}\cos\left(\pi -\alpha \right)\right]}^{\frac{3}{2}}-{\left[R^{2}+{\left(\frac{l}{2}\right)}^{2}-2R\cdot \frac{l}{2}\cos\alpha \right]}^{\frac{3}{2}}\right\}$$

An equation set, which correlates the filler’s location and its angle, before and after cellular growth, can then be written as follows:

(9) $$V_{1}=V_{2}$$

(10) $$V_{0}=V$$

By solving this equation set, the instantaneous angle and the location of any filler can be expressed as a function of its initial location, initial angle, cell size, and filler length.

### 2.2. Two-Cell model Approach

**Figure 2:**
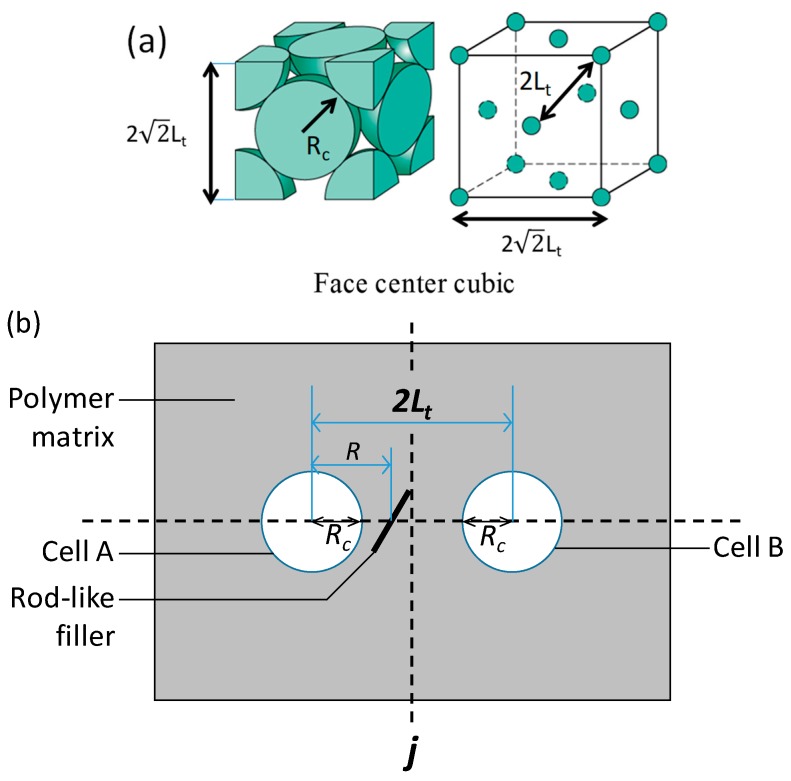
Illustrations of (**a**) the face centred cubic close pack model and (**b**) the rod-like fillers located on the centreline of two closest cells.

In a real situation, the rod-like filler will not be affected by only one cell. Two or even more growing cells may co-influence its location and angle. Therefore, we attempted to model the filler’s motion under the growth of two cells. To simplify the model, the fillers were assumed to be located at the centre line of the two cells. Even though this was a specialized general filler arrangement, it could still provide a straightforward way to analyze the co-influences of the multiple growing cells on the filler’s movement. In addition to the assumptions made in our model of one-cell growth, additional assumptions were made in the two-cell case: (1) The face-centred cubic (FCC) close pack model (Figure 2a) was used to describe the cell distribution in the polymer matrix; (2) In this model, the shortest centre-to-centre distance for two adjacent cells corresponded to the distance between the cell in the cube’s centre and the cell on the cube’s vertex that is, the 2*L*_t_ in Figure 2a, where the *L*_t_ was half of the distance between two cell centres; (3) Only fillers located on the centreline of these two closest cells were considered; (4) The filler movement was assumed to be affected by only two bubbles; (5) All of the cells grew simultaneously and were the same size at any time; (6) Once the cells had nucleated, all of the cell nucleation sites were fixed, and the cells grew without any relative displacement during the entire foaming process.

In the FCC close pack model, the cell density can be expressed as follows:

(11) $$\frac{4}{{\left(2\sqrt{2}\cdot L_{0}\right)}^{3}}=D$$

where 2*L*_0_ is the initial distance between the two closest cell nucleation sites at the beginning of the cellular growth, and *D* is the cell density. In one cube, the void fraction, β, can be expressed by Equation (12) as follows:

(12) $$\frac{4}{3}\times \pi \times R_{c}{}^{3}\times D=\beta$$

From Equations (11) and (12), we can express *R*_c_ as a function of *L*_0_ and β:

(13) $$R_{c}=L_{0}\times {\left(\frac{3\sqrt{2}\beta }{\pi }\right)}^{\frac{1}{3}}$$

**Figure 3:**
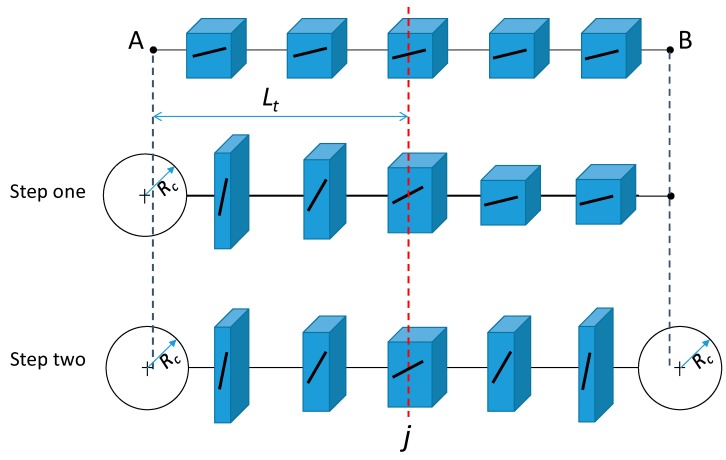
Illustration of two-step consecutive growth of Cells A and B used in modelling the growth of two cells.

Since a two-cell growth symmetrical effect was expected, we used a different coordinate. This is shown in Figure 2b, where the origin point is the middle point of the two cell centres. Thus, the negative and positive values of the filler’s location *R* represent the fillers close to Cell A and the fillers close to Cell B, respectively. At a specific cell density, the initial distance of the two neighboring cells can be calculated using Equation (11). Substituting the solved *R*_c_ with the known *L*_0_ into Equations (9) and (10), we can then obtain the instantaneous angle α and the location *R* of any filler from the growth of Cell A. To define the mutual and related effects of the growth of the two cells, we had to consider the second Cell B’s influence. We used a two-step cellular growth procedure to simulate the growth of two cells. As shown in Figure 3, in the polymer matrix around Cells A and B, at first Cell A started to grow to a radius of *R*_c_. Then, Cell B began to grow based on Cell A’s growth. Finally, we obtain the effects of the growth of two cells on filler movement. If we divide each step of cell growth into thousand sub-steps (that is, by dividing the growth of the *R*_c_ into thousand parts), we would confidently neglect the error found in the two-step method and obtain the pseudo-simultaneous growth of two cells. Using this strategy, we were able to estimate the functions between the final parameters and the initial status for two-cell growth. We used MATLAB (The MathWorks, Inc., Natick, MA, USA) to conduct all the calculations in this study.

## 3. Results and Discussion

### 3.1. Single Cell-Filler Interaction

**Figure 4:**
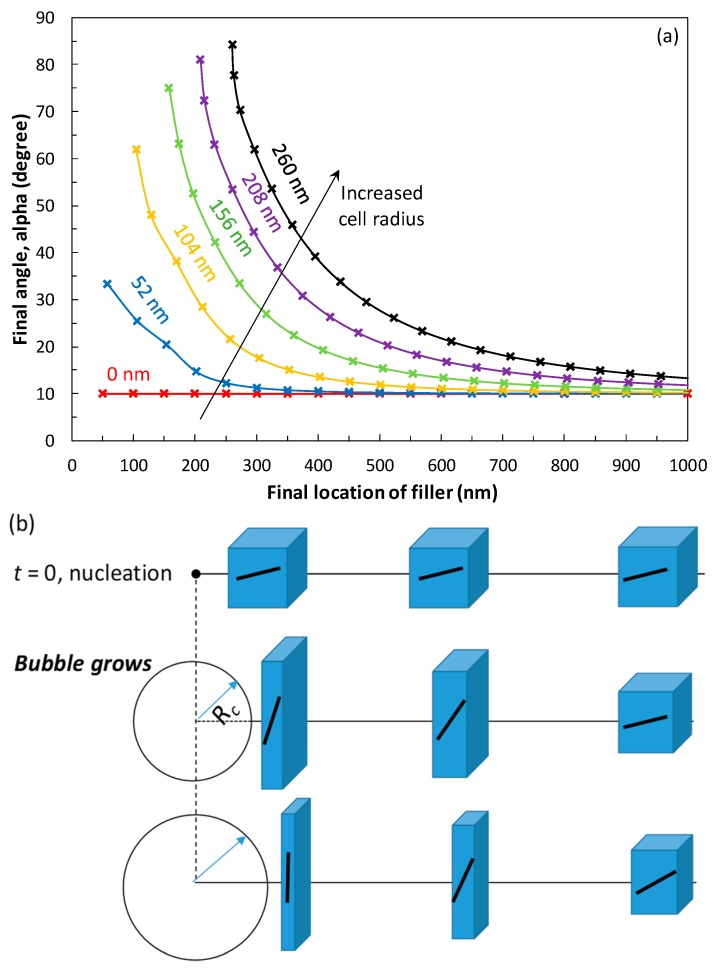
The effect of single cell growth on the filler orientation, expressed as (**a**) final angle, α vs. final location of filler; (**b**) schematic illustration of the filler’s rotational and translational displacements. Initial conditions: *l* = 260 nm, α_0_ = 10°.

Cellular growth in the foaming process can make the rod-like filler rotate and translate. The closer a filler is to the centre of a growing cell, the greater an impact it experiences. To quantitatively characterize the filler motion during cellular growth, we modelled the rotation and translation of one filler located around a single growing cell. Figure 4a shows the variation of the final radial angle, the α of a filler, with respect to its final location at various cell radii *R*_c_; that is, assuming a cell is growing near a rod-like filler with a length of 260 nm and an initial angle of 10°. *R*_c_ ranges from 0 to 260 nm. For a filler at a given distance from the cell nucleus centre, the changes in its angle and location are increased by an increased cell radius. For example, the filler initially located 50 nm away from the cell centre will move to 57.6 nm and will rotate from 10° to 33.3° when the cell radius is 52 nm. If the cell continues to grow up to 260 nm, the filler will then move 261 nm away from the cell centre and will rotate to 84°. In addition, as cell radius increases, the area affected by cell growth, as well as the fillers located in it, will be increased. It is obvious that, with a 52 nm radius, a cell can, at best, affect an area of around 300 nm, while a cell with a 260 nm radius can affect fillers located as far as 1000 nm away.

At a given cell radius, the impact of cellular growth on fiber motion deteriorates as the filler locates itself further away from the cell centre. The change in the final angle of the filler is decreased as the radial distance of the filler from the cell nucleus is increased. For example, in a 156 nm cell, the angle of the filler that was initially 0.4 nm away from the cell nucleus changed from 10° to 75°, but the filler that was initially 194 nm away from the cell only rotated to 20°. The same trend occurred in the translation of the filler. Both the rotation and translation trends of the filler indicated that the polymer domain closer to the cell had been more radially squeezed and bi-axially stretched. In Figure 4b, the schematic diagram shows the cell-growth-induced stretch or squeeze effect on the polymer matrix and how the filler rotation and translation was affected as a consequence of a single cell’s growth.

### 3.2. Two Cell-Filler Interactions

In a specific CPC foam with a fixed cell size, the impact of one single cell on the filler displacement is restricted to a limited area. The cell has little influence on the fillers that are far from it. In fact, cells will grow simultaneously in the polymer matrix at the locations of the cell nuclei. Thus, the fillers are also simultaneously affected by the growth of multiple cells. This makes it necessary to model the displacement of the fillers driven by the growth of multiple cells. Therefore, we also modelled the growth of two cells and then attempted to use the result to interpret the filler’s behaviour in the CPC foams during the cellular growth.

The motion of the rod-like fillers became far more complicated in relation to the growth of the two cells. To simplify the model, we studied only the fillers located on the line that connected the centres of the two cells. We also examined the fixed initial cell nucleation density from which the cell started to grow in each case. This was discussed later in this paper. We also studied the effects of several other factors; namely, the void fraction, the filler’s length, and the initial filler location and angle on its own movement during the cell’s growth.

#### 3.2.1. Effect of Initial Filler Location and Void Fraction

First, we modelled the final angle of the filler as a function of the final filler’s location at different void fractions, when the cell density was 2.54 × 10^14^ cells/cm^3^. We chose this number because a previous work [42] had shown that the optimal percolation threshold of the CPC foams’ conductivity occurred when the cell size was ~100 nm, cell density was ~2.54 × 10^14^ cells/cm^3^, and the void fraction was ~30%. Thus, it was particularly important to investigate the filler motion at that cell density. At a fixed cell density, the cell radius is fixed once the void fraction is determined. Therefore, like the one-cell growth case, the two-cell-filler interaction of the cell radii at 0, 47, 62, 74, 86, and 98 nm, which corresponded to the void fractions at 0, 0.1, 0.2, 0.3, 0.4, and 0.5, respectively, were simulated using the proposed two-cell growth model. The filler length *l* and the initial filler angle α_0_ were set at 260 nm and 10°, respectively, so as to be comparable with the one-cell growth case.

**Figure 5:**
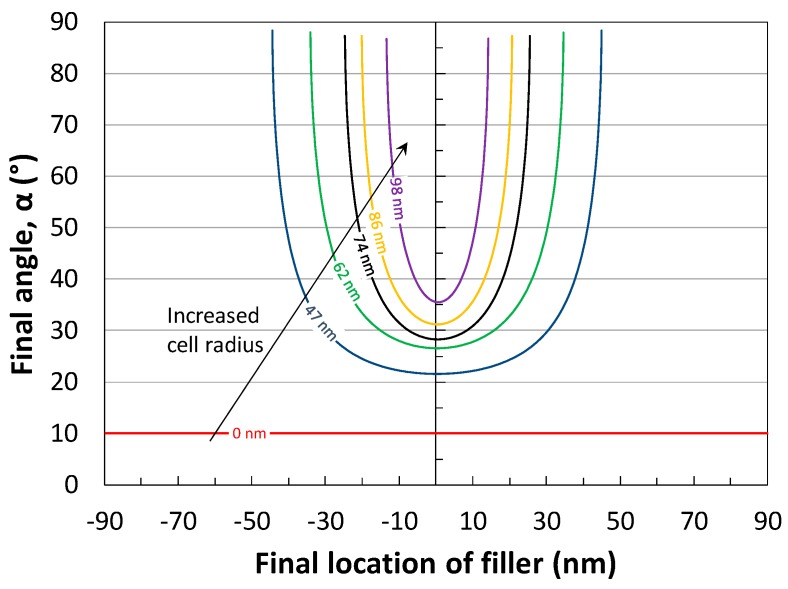
Effect of two-cell growth on the final angle of filler located in the cell centreline. Cell growth is simulated using the two-step consecutive growth of cells (Figure 4). Final angle vs. final location of filler at five different expansion ratios (i.e., five cell radii) was illustrated. *l* = 260 nm, α_0_ = 10°, and *D* = 2.54 × 10^14^ cells/cm^3^, cell size increases from 0 to 98 nm (void fraction increases from 0 to 0.5).

The results in Figure 5 show the effects of the two-cell growth on the filler’s rotation and translation. At the beginning of the cellular growth, along the line connecting the two cell centres, the final filler angle decreased as the filler distance increased from one cell centre to the other. This reached its lowest value at the midpoint of the line connecting the two cell centres. Then, it increased, as the filler location approached the second cell’s centre, and it presented a symmetrical pattern. This was logical because the two cells on either side of the filler had equally influenced the filler’s motion. The filler at the axis of the symmetry had the lowest final angle because it was located the farthest away from both cells. The translation of the filler showed the same symmetrically changing trend as the filler rotation with respect to the distance of the filler from the cell centres. At the midpoint, the filler did not move at all. This was because it experienced squeezing pressures that were of the same magnitude, but which came from opposite directions; thus, the net force was zero. As the filler distance decreased in relation to either cell centre, it exhibited a greater degree of displacement.

As the void fraction increases (via an increase in the cell radius *R*_c_), the polymer between the two cells is radially squeezed and bi-axially stretched. Consequently, the forces exerted on the fillers in between will increase, and this will increase both the filler’s rotation and its translation. For example, at the midpoint, the filler’s angle increased from the initial 10° to about 35° when the cell grew to 98 nm, but only to 21° when the cell grew to 47 nm. With cell growth, the fillers that were initially evenly distributed between the cell nucleation centres were then squeezed into the narrowed areas in between two cells.

#### 3.2.2. Effect of the Initial Filler Angle

**Figure 6:**
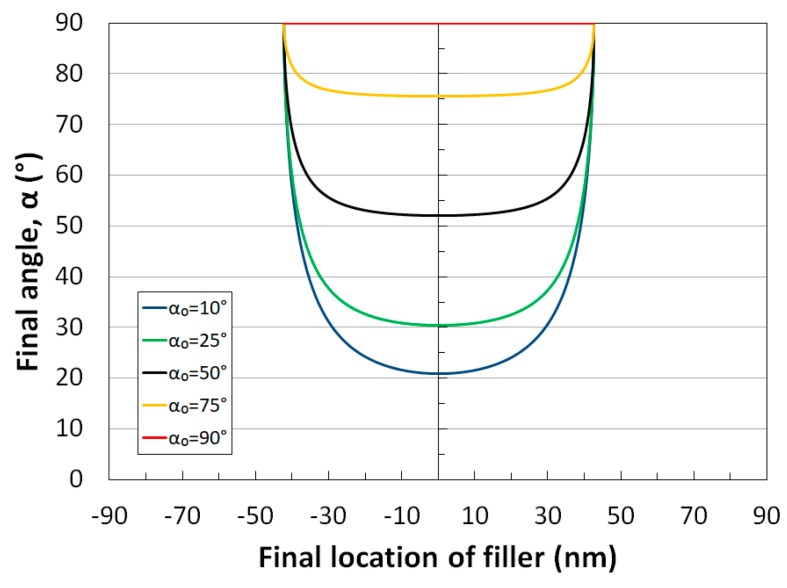
The rotation of filler at different initial angle during the two-cell growth. *l* = 260 nm, β = 0.1, *D* = 2.54 × 10^14^ cells/cm^3^, and *R*_c_ = 47 nm.

To better understand the filler’s rotation during cell growth, its rotation at a different initial filler angle α_0_ at a given void fraction was also modelled and is shown in Figure 6. The α_0_ varies from 10° to 90°. The fillers became harder to rotate as their initial angles were increased. In other words, the fillers with smaller angles were more sensitive to the cellular growth. If the filler is initially perpendicular to the cell centre-centre line (i.e., α_0_ = 90°), it cannot be rotated at all. This indicates that the fillers randomly distributed in the polymers will be aligned so as to be tangential to the cellular radii. For practical purposes, the alignment of the fillers must be controlled to a certain extent, so as to keep the fillers in contact with each other as much as possible.

#### 3.2.3. Effect of the Relative Size of the Filler and the Cell

The cell size could be different under the same void fraction (that is, the cell density can be different). This means that the electrical conductivity or the filler’s interconnectivity might not be increased, even with a proper void fraction if the cell size, relative to the filler’s length, was not also optimal. On the other hand, the filler’s length also plays a critical role in its re-orientation during cellular growth. A shorter filler relative to the cell size is easier to rotate. Therefore, it was also important to investigate the effect of the relative size of the filler and the cell on the filler’s motion.

**Figure 7:**
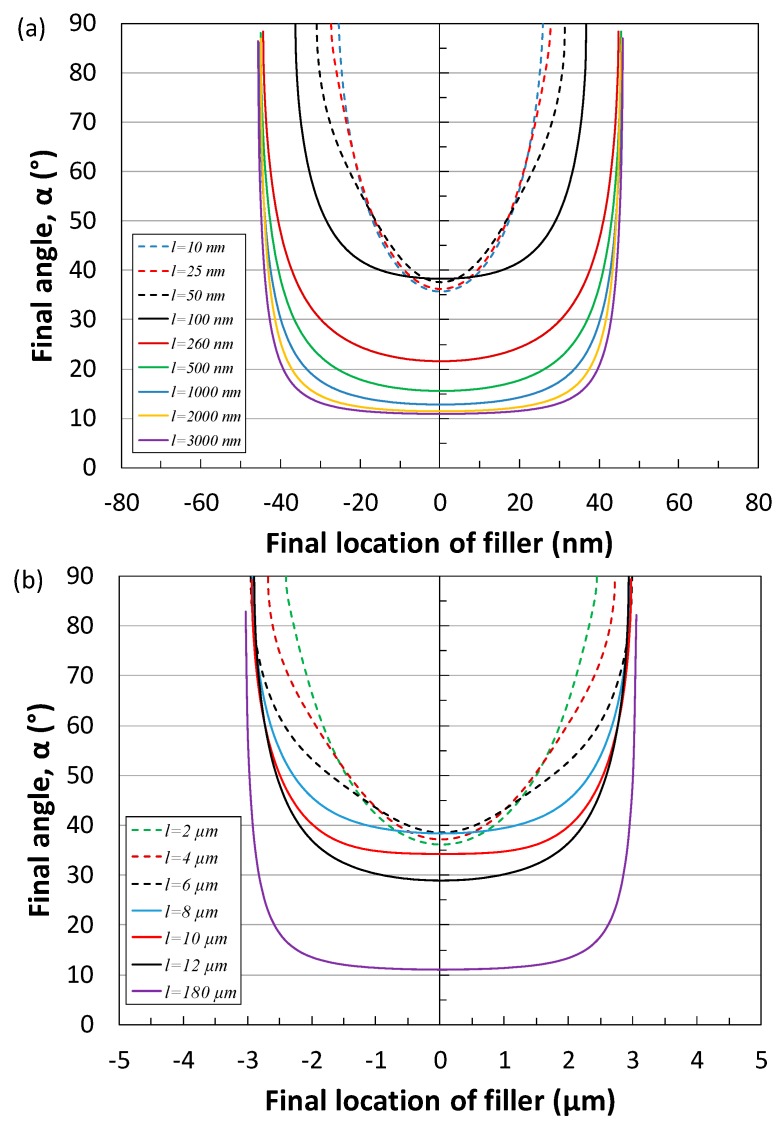
Effect of filler length and cell size on rotation of filler when the initial angle is 10°. (**a**) Nanocellular, β = 0.1, *D* = 2.54 × 10^14^ cells/cm^3^, *R*_c_ = 47 nm; (**b**) microcellular, β = 0.1, *D* = 9 × 10^9^ cells/cm^3^, *R*_c_ = 3 μm.

To quantify the effect of the relative size of the filler and the cell, we simulated the filler rotation under different filler lengths in two representative cell structures (that is, nano- and micro-cellular foams). As shown in Figure 7a, in the nanocellular foam, when the filler length was smaller than the cell size or just comparable to it (that is, equal to or 2-fold larger), the filler located around the midpoint, where there was the least rotation, had rotated from 10° to about 35°. This showed that a short filler was easier to rotate. The filler rotation was more difficult when the filler length was increased. When the filler length was about 60 times larger than the cell radius, the fillers in between barely rotated, except those located very close to the cells. A similar effect of the filler’s length on its rotation in microcellular foams is shown in Figure 7b. These results indicate that an appropriate filler length relative to the cell size is vitally important in preparing CPC foams with the desired electrical conductivity.

### 3.3. Illustration of the Two-Cell Growth

**Figure 8:**
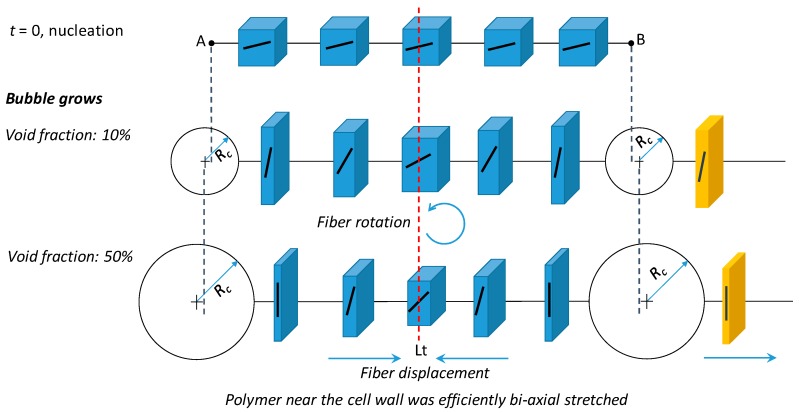
Schematic illustration of how the growth of two cells influence the fiber orientation and location. Fillers located in between the two cells and on the other side of one cell are both shown.

The effect of the two cells’ growth on the filler’s orientation and location is shown in a schematic diagram in Figure 8. At *t* = 0, when the bubbles were under the nuclei, all the fillers at different locations on the line connecting the two-cell centres were assumed to have the same initial angle. Once the bubbles started to grow, the polymer near the cells was efficiently bi-axially stretched. As a result, the filler rotated toward the same direction, which, in this case, was counter-clockwise. As the void fraction β increased, the filler continuously rotated, and the filler to filler distance was squeezed. Additionally, the fillers near the cells tended to become perpendicular to the cell-to-cell line. Moreover, at a fixed void fraction, the polymer close to the cells had more deformation than the polymers around the midpoint. Hence, the fillers in the polymer matrix next to the cells had more movement (both rotation and translation) than the fillers in the polymers close to the midpoint.

**Figure 9:**
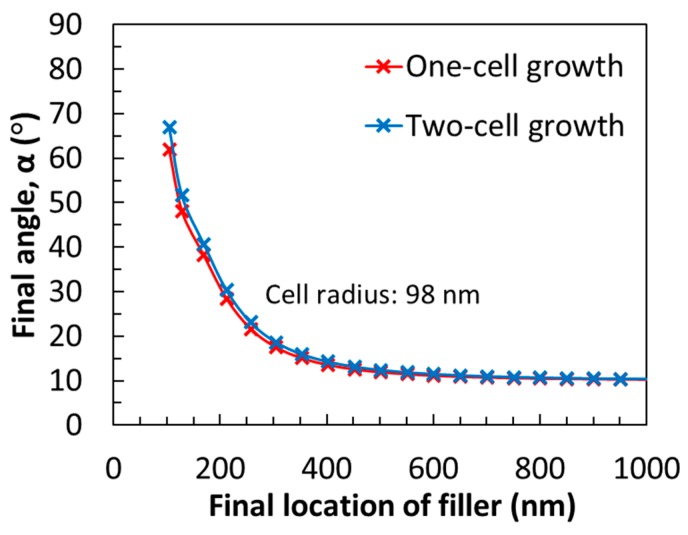
Filler movement under one-cell growth and two-cell growth with fillers located on the other side of cells rather than in between the two cells (illustrated in Figure 8).

We only considered the fillers located in between the two cells. This was because each cell would only affect the fillers located close to it, but would not affect the fillers located further away. Hence, Cell A, as shown in Figure 8, would not affect the movement of the fillers located on the right side of Cell B. The fillers located on the right side of Cell B, rather than being located in between the two cells, were too far away from Cell A to receive any significant impact from it. In Figure 9, the one-cell case was compared with the two-cell case, where the fillers were on the right side of Cell B. The filler movement curve in the one-cell case almost completely overlapped the same curve in the two-cell case. This indicated that Cell A had not affected the filler rotation and translation at all. This observation theoretically supported the idea that the current two-cell growth model could also be used to describe the multi-cell growth system in all CPC foams.

### 3.4. Experimental Verification

**Figure 10:**
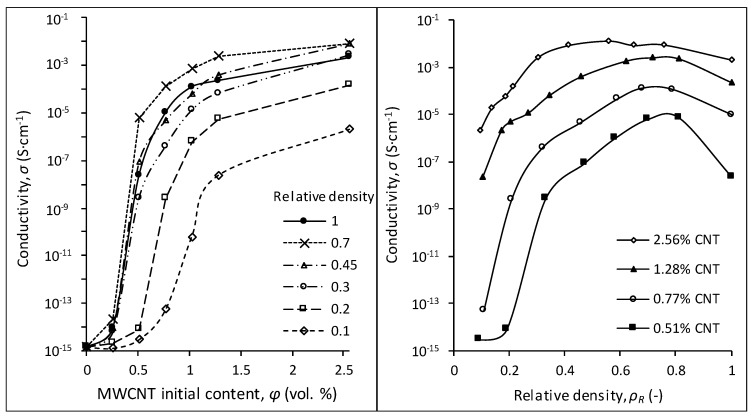
Electrical conductivity of PP/MWCNT nano/microcomposite foams as a function of (**a**) different MWCNT initial content and (**b**) relative density [42].

We compared our theoretically predicted results with our earlier experimentally observed PP/MWCNT data [42]. Our previous experiments showed that, at a relative density of around 0.7 (that is, a void fraction of 0.3), the PP/MWCNT nanocomposite foam’s electrical conductivity was enhanced and was even higher than in the solid sample (Figure 10) for all of the CNT content. Du et al. [43] and Gong et al. [44] both proposed that this conductivity enhancement (that is, a decreased percolation threshold) resulted from the slight CNT alignment. Chang et al. [45] also obtained similar results using a Monte Carlo simulation to estimate the percolation threshold of fillers with different filler orientations. It was found that the percolation threshold was minimized when the fillers were slightly oriented (both uniaxially and biaxially) rather than being completely isotropic. This corresponded with the modelling results in our study, which had shown that the conductive fillers were re-oriented during cell growth and that a relatively low void fraction caused a slight filler orientation and relocation. We thus hypothesized that the slight orientation of the conductive fillers at a proper void fraction in CPC foams can increase their conductivity and decrease their percolation threshold. More research is required to relate the filler interconnectivity (that is, the electrical conductivity) to foaming actions.

## 4. Conclusions

There is an absence of any quantitative analysis of the rotation and translation of conductive fillers due to cell growth in CPC foams. This includes those factors that would influence the filler’s orientation and location. Although our modelling is simple and many assumptions are made, this study is a first attempt to initiate a discussion about this knowledge gap. The analytical model we developed successfully describes the instantaneous rod-like filler rotation and translation during the cellular growth in CPC foams. Our modelling results show that, as a cell grows, its affected polymer area also grows. The filler located in the polymer thus has more movement. Besides the void fraction, the relative size of the filler and cell, as well as the initial filler location and angle, has a substantial impact on the filler’s orientation and translation.
